# Expression of calcitonin gene-related peptide in the infrapatellar fat pad in knee osteoarthritis patients

**DOI:** 10.1186/s13018-017-0568-1

**Published:** 2017-04-21

**Authors:** Jun Aikawa, Kentaro Uchida, Shotaro Takano, Gen Inoue, Atsushi Minatani, Masayuki Miyagi, Dai Iwase, Hiroyuki Sekiguchi, Manabu Mukai, Masashi Takaso

**Affiliations:** Department of Orthopedic Surgery, Kitasato University School of Medicine, 1-15-1 Minami-ku Kitasato, Sagamihara, Kanagawa 252-0374 Japan

**Keywords:** Calcitonin gene-related peptide, Knee osteoarthritis, Infrapatellar fat pad

## Abstract

**Background:**

The infrapatellar fat pad (IPFP) has been implicated as a possible source of osteoarthritis (OA) development and knee pain due to the production of inflammatory mediators and the existence of nerve fibers within this structure. Calcitonin gene-related peptide (CGRP) is a vasodilatory neuropeptide that is localized to joint tissues and has recently been implicated in the development of knee OA and OA pain. To date, however, the expression levels of CGRP in the IPFP of human knee OA patients have not been examined.

**Methods:**

IFFP and synovial (SYN) tissues were harvested from 100 individuals with radiographic knee OA (unilateral Kellgren/Lawrence [K/L] grades 2–4) during total knee arthroplasty and subjected to immunohistochemical analysis for CGRP localization. In addition, the messenger RNA (mRNA) expression levels of CGRP and cyclooxygenase-2 (COX-2) in the collected tissues were evaluated and compared using real-time PCR analysis of total RNA extracts. CGRP and COX-2 mRNA expression were also compared among individuals with K/L grades 2–4.

**Results:**

CGRP-positive cells were detected in the capillaries within the IPFP and lining layer of SYN tissue. The expression levels of CGRP in the IPFP were positively correlated with COX-2 and were significantly higher than those in SYN tissue. CGRP expression in tissue from the KL4 group was twofold higher than that from the KL2 group.

**Conclusions:**

The IPFP of knee OA patients produces relatively high levels of CGRP, which may be regulated by COX-2 at the transcriptional level. Further studies are needed to determine if CGRP levels are directly linked to OA pathology.

## Background

Knee osteoarthritis (OA) patients often present with cartilage degeneration and joint pain that ranges from moderate to severe. Evidence suggests that several joint tissues, including synovial (SYN) tissue [1–3], joint capsule [4], and menisci [5–7], are involved in the development of OA and OA pain. Despite these findings, the mechanisms underlying OA pathology are not fully understood.

The infrapatellar fat pad (IPFP), which is located at the anterior aspect of the knee and is surrounded by the SYN membrane, may contribute to OA development or pain [8, 9]. The IPFP also secretes a number of pro-inflammatory cytokines, including tumor necrosis factor-alpha (TNF-α) and interleukin-6 (IL-6), that contribute to OA development [10–12]. Further, the IPFP is particularly sensitive to pain and is richly innervated by nerve fibers originating from the posterior tibial nerve [13]. These nerve fibers include peptidergic C-fibers, which are nociceptive and produce a number of neuromodulatory substances [14]. In addition, patients who experience knee pain after total knee arthroplasty often have increased numbers of sensory nerves in the IPFP [15], suggesting that the IPFP serves as a terminus for sensory nerve innervation in the knee joint. Therefore, a better understanding of the expression and regulation of OA-associated molecules in the IPFP may reveal the contribution of this structure to OA development and knee pain.

Among the molecules found in joint tissue is the vasodilatory neuropeptide calcitonin gene-related peptide (CGRP), which binds to the calcitonin receptor-like receptor (CLR) and receptor activity-modifying protein 1 (RAMP1) [16]. The levels of CGRP in several human joint tissues are associated with the intensity of OA pain and OA development [4, 5, 17–19]. For example, CGRP-positive nerve fibers are increased in the menisci and joint capsule of knee and hip OA patients, respectively [4, 5]. CGRP expression is also increased in SYN of knee OA patients, implicating the involvement of this molecule in inflammation, OA development, and OA pain [17–19]. To date, however, CGRP expression in the IPFP has not been investigated in human OA patients.

Here, we attempted to determine the localization and expression levels of CGRP in the IPFP of 100 individuals with knee OA. CGRP expressions levels in the IPFP were also compared to those in the SYN to investigate whether IPFP is a major source of CGRP in the joint tissue of OA patients.

## Methods

### Patients

Power analysis using an alpha of 0.05 and power of 0.80 was conducted in G*POWER3 to determine a sufficient sample size. Power analysis revealed that 64 samples for CGRP and 97 samples for cyclooxygenase-2 (COX-2) were needed to detect a difference between SYN and IPFP. Therefore, data were collected from 100 participants. The study population consisted of 23 men and 77 women (age: range = 46–89 years, mean ± standard deviation (SD) = 73.3 ± 0.8 years; body mass index (BMI): range = 18.2–36.7, mean ± SD = 25.8 ± 0.4 kg/m^2^) with radiographic knee OA (unilateral Kellgren/Lawrence [K/L] grades 2 (2/100, 2%), 3 (40/100, 40%), and 4 (58/100, 58%)). All study participants underwent total knee arthroplasty at our institution between March 2015 and January 2017. Informed consent for participation in this study was obtained from each patient on the day before surgery. During the surgery, samples of IPFP and SYN were harvested from each operated knee. A portion of each IPFP and SYN sample was immediately frozen in liquid nitrogen at −80 °C until needed for RNA extraction. The remaining tissue was fixed in 4% paraformaldehyde formalin for 48 h for histological analysis.

### Immunohistochemistry

The paraformaldehyde-fixed IPFP and SYN samples were embedded in paraffin, sliced into 3-μm-thick sections, and deparaffinized using xylene. The sections were reacted with primary mouse monoclonal primary antibody against CGRP (Abcam) and rabbit polyclonal primary antibody against COX-2 (Abcam, Cambridge, MA) for 6 h at 4 °C. The sections were further incubated with Alexa Fluor 488 goat anti-rabbit IgG and Alexa Fluor 594 goat anti-mouse IgG secondary antibodies (Thermo Fisher Scientific, Waltham, MA, USA) for 1 h at room temperature. Sections incubated in serum containing no primary antibody were used as negative controls. The fluorescence-stained sections were visualized using fluorescence microscopy (Axiovert 200, Zeiss, Jena, Germany).

### Real-time PCR analysis

Total RNA extraction from IPFP and SYN and cDNA synthesis were performed as described previously [18]. PCR primer pair sequences used for real-time PCR analysis were as follows: CGRP-FWD (5′-TTGCCCAGAAGAGAGCCTGTG-3′) and CGRP-REV (5′-TTGTTCTTCACCACACCCCCTG-3′) for CGRP amplification; COX-2-FWD (5′-TGGCTGAGGGAACACAACAG-3′) and COX-2-REV (5′-AACAACTGCTCATCACCCCA-3′) for COX-2 amplification; and GAPDH-FWD (5′-TGTTGCCATCAATGACCCCTT-3′) and GAPDH-REV (5′-CTCCACGACGTACTCAGCG-3′) for GAPDH amplification. Melt curve analysis was used to examine the specificity of the amplified products. The relative messenger RNA (mRNA) expression levels of CGRP and COX-2 were examined by quantitative PCR using the CFX-96 Real-Time PCR Detection System (Bio-Rad, CA, USA). CGRP and COX-2 mRNA expression was normalized to that of the house keeping gene, GAPDH. Further, CGRP and COX-2 mRNA expression levels were compared between IPFP and SYN, and among individuals with K/L grades 2 to 4.

### Statistical analysis

Differences in CGRP and COX-2 expression between IPFP and SYN tissues were compared using the paired *t* test. The relationship between CGRP and COX-2 expression was evaluated using Pearson’s correlation coefficient. Potential statistical outliers that may have influenced the linear regression coefficient analysis were identified using Cook’s distance statistical test. All statistical analyses were performed using SPSS software (v. 19.0; SPSS, Chicago, IL, USA). A *p* value of <0.05 was considered statistically significant for all analyses, with the exception of the linear regression coefficient analysis, for which a *p* value of <0.01 was considered statistically significant.

## Results

### Localization of CGRP in the IPFP and SYN of OA patients

**Fig. 1:**
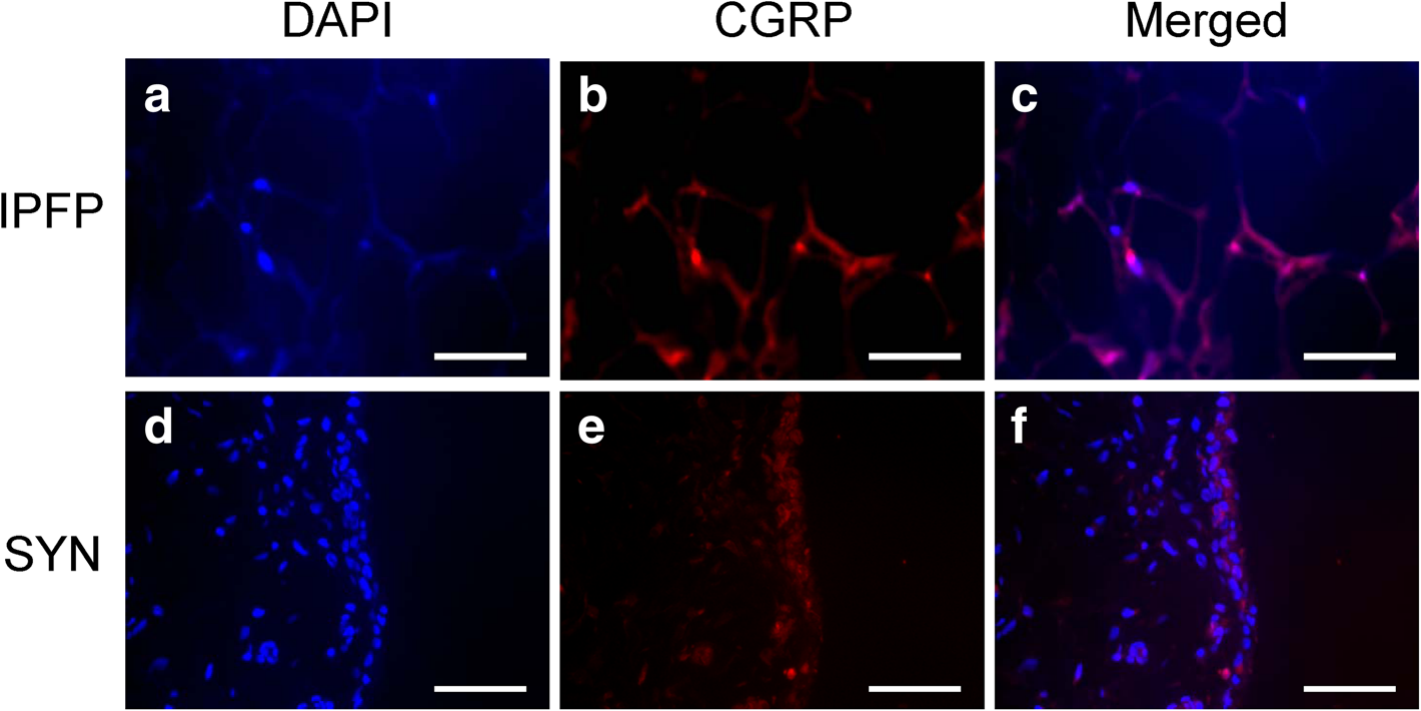
Immunostaining of CGRP in the infrapatellar fat pad and synovial tissue of knee OA patients. Infrapatellar fat pad (**a**–**c**) and synovial tissue (**d**–**f**) stained with (**a**, **c**) DAPI (nuclei) or (**b**, **d**) CGRP. **c**, **f** The merged images. *Scale bar* = 100 μm

Immunohistochemical analysis was performed to investigate the localization of CGRP in the IPFP tissue of OA patients removed during arthroplastic surgery (Fig. 1a–c). SYN was included for comparative analysis (Fig. 1d–f). Immunostaining revealed that CGRP protein was localized to the capillaries of IPFP (Fig. 1a–c) and the lining layer of the synovium (Fig. 1d–f). No immunostaining was observed in negative control sections.

### Expression of CGRP and COX-2 in IPFP and SYN of OA patients

**Fig. 2:**
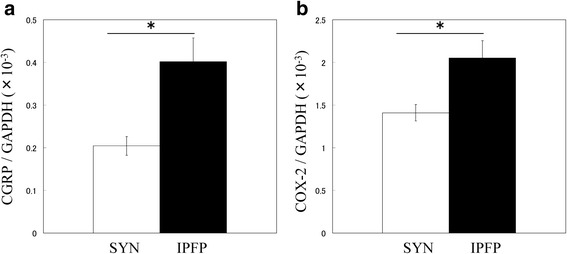
Real-time PCR analysis of CGRP and COX-2 mRNA expression in the infrapatellar fat pad and synovial tissue of knee OA patients. **a** CGRP and **b** COX-2 mRNA expression in the infrapatellar fat pad and synovial tissue of knee OA patients. *Statistically significant difference between IPFP and SYN (*p* < 0.001). All data are presented as the mean ± standard error (*n* = 100)

The expression of CGRP and COX-2 in the IPFP and SYN of knee OA patients was compared by RT-PCR (Fig. 2). CGRP expression was significantly higher in IPFP than in SYN (Fig. 2a). COX-2 expression was also significantly higher in IPFP compared to SYN (Fig. 2b).

### Relationship between CGRP and COX-2 expression levels in the IPFP of OA patients

**Fig. 3:**
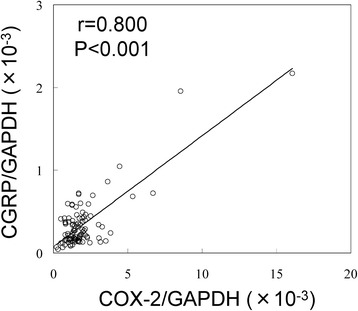
Correlation between CGRP and COX-2 mRNA expression levels in the infrapatellar fat pad and SYN tissues of knee OA patients. Correlation between CGRP and COX-2 mRNA expression levels in IPFP tissues harvested from 99 knees of OA patients. The values for one IPFP were removed as outliers

A correlation between CGRP and COX-2 expression levels was previously identified in the synovium of OA patients [18]. In addition, prostaglandin E2 (PGE2), the enzymatic product of COX-2, was shown to stimulate synovial CGRP gene expression [18]. To investigate the possible regulation of CGRP by COX-2 in IPFP, CGRP and COX-2 expression levels in the IPFP of OA patients were measured (Fig. 3). One IPFP was an outlier for CGRP expression and was therefore excluded from the analysis. Based on the results of the expression analysis, the levels of COX-2 mRNA were positively correlated with those of CGRP in IPFP (Fig. 3).

### Correlation of CGRP and COX-2 expression among K/L grades

**Fig. 4:**
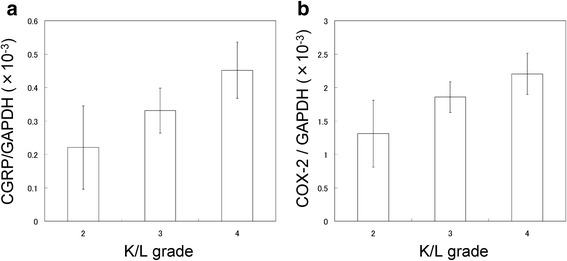
Relationship between CGRP and COX-2 mRNA expression level and K/L grade. **a** CGRP and **b** COX-2 mRNA expression in the infrapatellar fat pad of knee OA patients. All data are presented as the mean ± standard error

To investigate the relationship between CGRP and COX-2 expression among K/L grades of OA, we analyzed CGRP and COX-2 expression in individuals with K/L grades 2 to 4. CGRP expression was 1.5- and 2.0-fold higher in individuals with K/L grades 3 and 4 compared to those with K/L grade 2, respectively (Fig. 4a). COX-2 expression was 1.4- and 1.7-fold higher in individuals with K/L grades 3 and 4 compared to those with K/L grade 2, respectively (Fig. 4b).

## Discussion

In the IPFP of knee OA patients, CGRP-expressing cells were observed in the capillaries of adipose tissue. Notably, the expression of CGRP and COX-2 in the IPFP was higher than that in the SYN tissue, although a positive correlation between CGRP and COX-2 expression was detected in both tissues. These findings demonstrate that the IPFP is a source of CGRP in the joint tissue of OA patients.

Previous studies reported that CGRP-positive cells are present in the synovial lining layer of developmental dysplasia of the hip and OA patients [18, 20]. Consistent with previous reports [18], CGRP was localized in the synovial lining layer of knee OA patients. CGRP-positive cells were also detected in the capillaries of the IPFP of knee OA patients. Notably, CGRP expression in the IPFP was significantly higher than that in the SYN. These results suggested that IPFP may be a source of CGRP production in joint tissues.

The IPFP secretes several inflammatory cytokines, including TNF-α and IL-6, at markedly higher levels compared to autologous subcutaneous adipose tissue (SCAT) [11, 12]. CGRP expression is regulated by TNF-α, IL-1β, and PGE2 in several cell types, such as synovial [18], immune [21–23], epithelial [24], and neural cells [25, 26]. PGE2 has been shown to stimulate CGRP gene expression in cultured synovial cells [18]. Here, a correlation between CGRP and COX-2 expression was also detected in the IPFP harvested from knees of OA patients, suggesting that the COX-2 pathway may regulate CGRP expression in the IPFP.

The elevation of CGRP levels in OA patients has been suggested to lead to OA development or increased OA pain [4, 5, 17]. Serum levels of CGRP in OA patients are significantly correlated with K/L grade and pain score [17]. Here, CGRP expression in the IPFP of KL grade 4 individuals was 2-fold higher than in that of KL2 patients. CGRP in IPFP may contribute to OA development. However, our study lacked sufficient numbers of KL grade 2 patients for statistical analysis and a healthy control group. Further investigation is needed to reveal the contribution of CGRP to OA pathology.

Several limitations of the present study warrant mention. First, because a non-OA patient population was not included in the study, further experiments are needed to confirm whether CGRP levels are elevated in the IPFP of OA patients compared to non-OA patients. Second, although CGRP levels were higher in IPFP than SYN tissue, determining the presence of a direct causative link between CGRP and pain requires evaluation of the relationship between pain score and CGRP level. Third, in vitro studies using adipocytes and stromal/vascular cells are required to confirm if CGRP is regulated by COX-2/PGE2 in adipose tissue. Finally, the concentration of CGRP protein in IPFP was not determined.

## Conclusions

The IPFP of knee OA patients produces relatively high levels of CGRP, which may be regulated by COX-2 at the transcriptional level. Further study is required to determine the contribution of CGRP in IPFP to OA pathology.

## Abbreviations

CGRP: Calcitonin gene-related peptide
CLR: Calcitonin receptor-like receptor
IPFP: Infrapatellar fat pad
IL-6: Interleukin-6
OA: Osteoarthritis
PGE2: Prostaglandin E2
RAMP1: Receptor activity-modifying protein 1
SYN: Synovial tissue
TNF-α: Tumor necrosis factor-alpha
